# Percutaneous atrial septal defect occlusion through thrombosed inferior vena cava filter

**DOI:** 10.1002/ccr3.4350

**Published:** 2021-08-24

**Authors:** Radotseheno Rolland Rakotonoel, Gilles Rioufol, William Uhlrich, François Derimay

**Affiliations:** ^1^ Cardiovascular Hospital and Claude Bernard University and Univ Lyon CarMeN Laboratory INSERM INRA INSA Lyon Université Claude Bernard Lyon 1 Lyon France; ^2^ Cardiology Unit Joseph Raseta Befelatanana University Hospital Antananarivo Madagascar

**Keywords:** atrial septal defect, IVC filter, mini TEE

## Abstract

Thrombosed inferior vena cava (IVC) should not be considered as a limitation to femoral access for cardiac structural procedures. Reopening by angioplasty in the same procedural step is feasible and safe.

## Graphical abstract

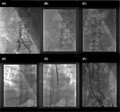

## 1 INTRODUCTION

We report percutaneous atrial septal defect (ASD) occlusion through a thrombosed inferior vena cava filter. This demonstrates the technical feasibility and safety of combined vena cava reopening and ASD occlusion in the same step. Thrombosed inferior vena cava (IVC) filter seems not to be a contraindication for femoral access for cardiac structural treatment.

Indications for atrial septal defect (ASD) occlusion can be hemodynamic (right ventricular dilatation by volume overload in absence of pulmonary arterial hypertension) or for paradoxical embolism, to prevent stroke recurrence.^1^ When the edges are adequate, percutaneous treatment is the favorite strategy for ostium secundum ASD closure, applied in 80% of ASDs.^1^, ^2^ The anatomy of primum ASD or sinus venosus, however, does not provide enough edge for good stability and can be associated with other cardiac abnormalities. Venous femoral access is then the only option. The present case report describes, for the first time, percutaneous ASD occlusion through a totally thrombosed IVC filter 5 months after implantation in acute‐phase stroke.

## 2 CASE REPORT

**FIGURE 1:**
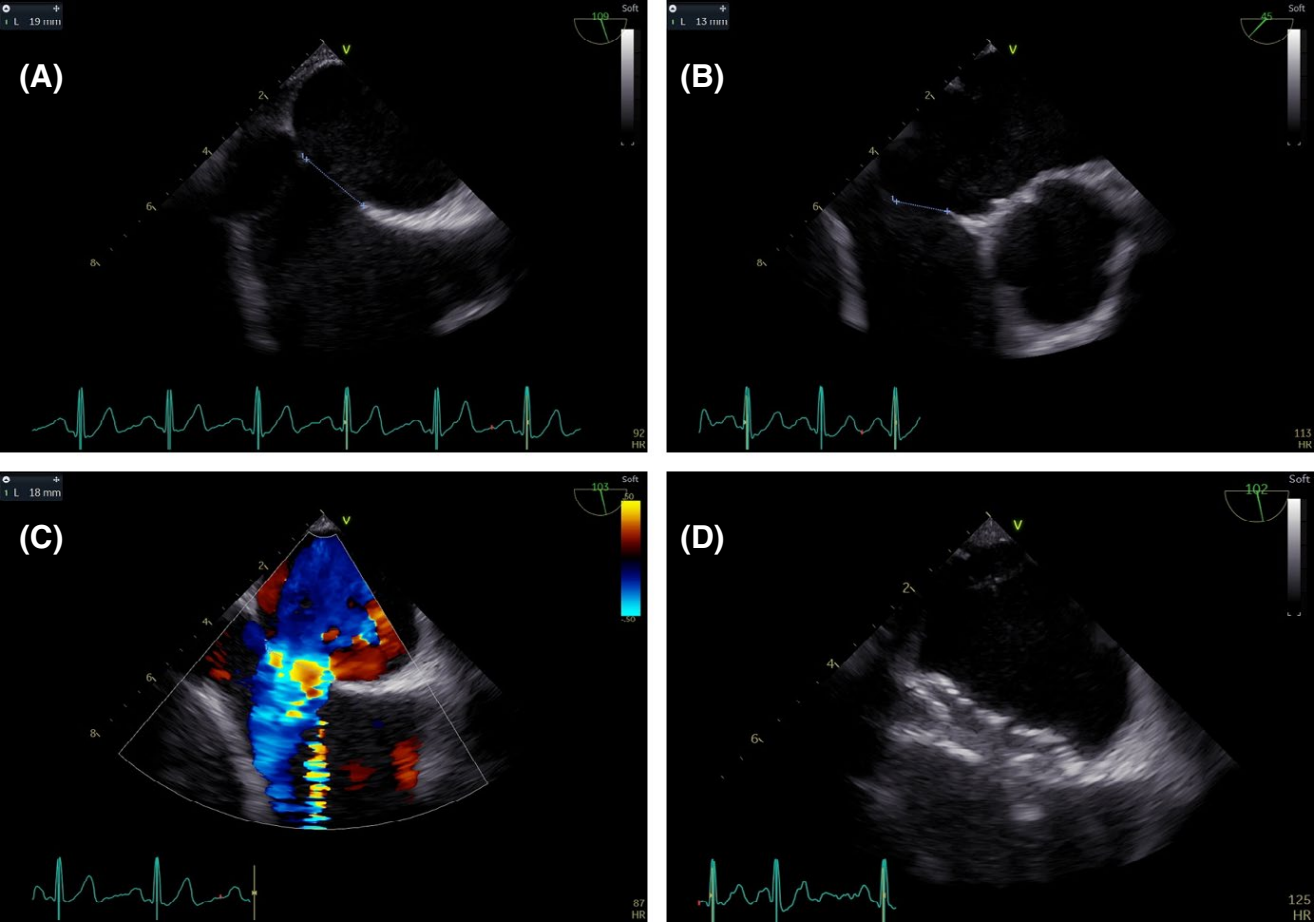
Procedural TEE acquisition. A and B, maximum and minimum diameters of ostium secundum ASD. C, left‐right unidirectional shunt. D, final control after 24 mm ASD prosthesis implantation (Figulla Occlutech, Germany)

A 47‐year‐old man with familial polycystic kidney disease presented with left sylvian ischemic stroke, with complete recovery after thrombolysis. The culprit mechanism was identified as paradoxical embolism after discovery of ASD associated with proximal bilateral phlebitis; no other etiologies were identified. A large thrombophilic check‐up identified no real factors. The ASD was of the ostium secundum type, size 19 × 13 mm on transesophageal echocardiography (TEE) (Figure 1A and B). Due to absolute contraindications against all anticoagulation treatments (VKA, DOACs, heparin), given the macroscopic hematuria associated with polycystic kidney disease, a vena cava filter was implanted in the acute phase (ALN, France). Patient was discharged from neurology at day 7.

Five months poststroke, ASD occlusion was decided on as secondary prevention. On TEE, edges were compatible with a percutaneous occlusion strategy, avoiding more complex surgical closure. TTE confirmed the absence of pulmonary arterial hypertension. The right ventricle was slightly dilated but normo‐kinetic, without other cardiac abnormalities. Before the procedure, complete IVC occlusion upstream of the vena cava filter was observed on Doppler.

**FIGURE 2:**
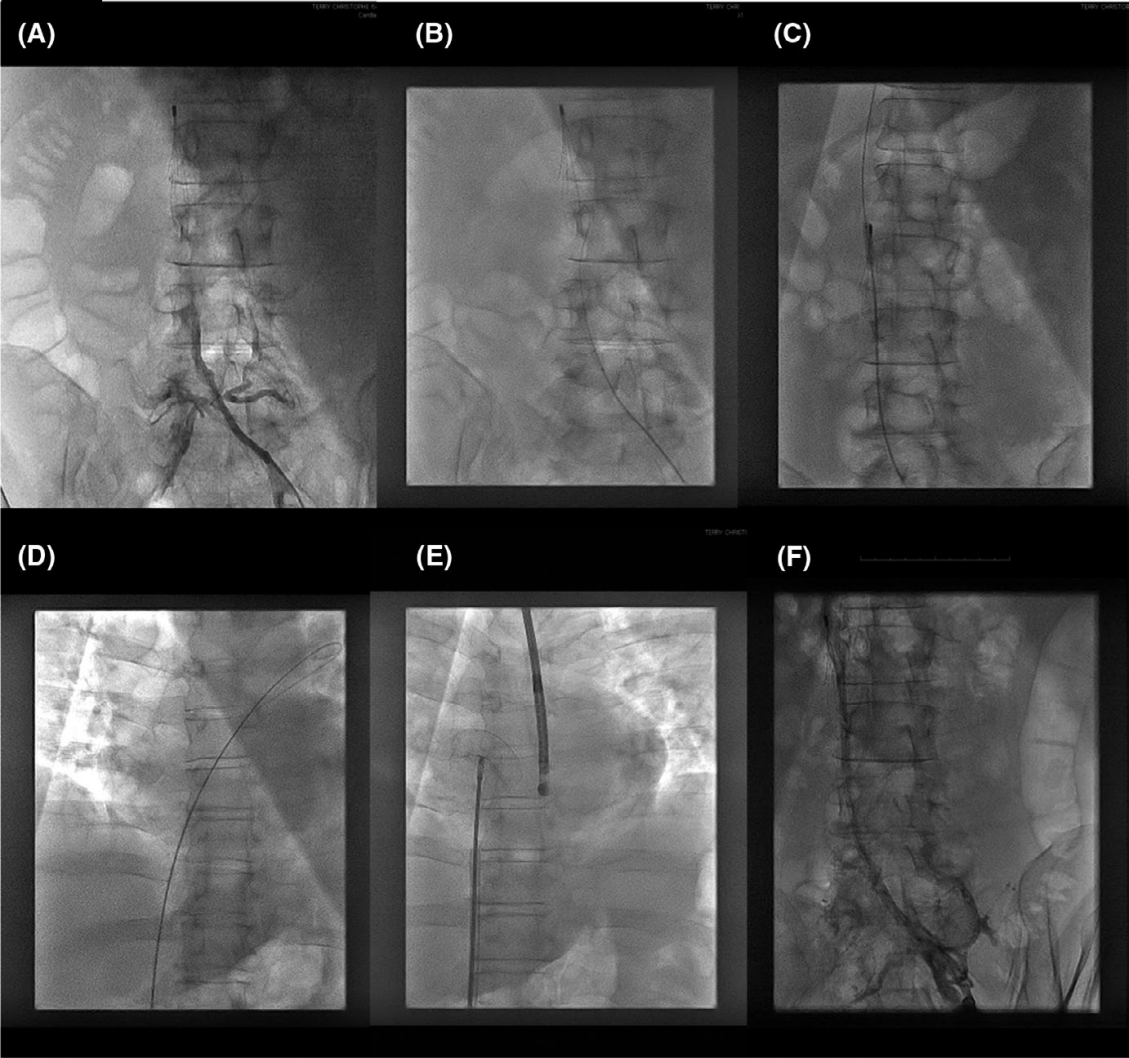
inferior vena cava (IVC) reopening and ASD closure. A, total occlusion of IVC. B, occlusion crossing. C, reopening by 5.0 mm balloon angioplasties inside vena cava filter. D: 0.035 mm wire positioning in left superior pulmonary vein through ASD. E, ASD prosthesis deployed. F, final IVC angiographic control without rupture

The procedure was performed under local anesthesia, guided by TEE and X‐ray. The TEE device was a pediatric mini probe (General Electric). A 12‐French desilet was positioned under echo guidance in the left femoral vein (right side thrombosed). Nonfractionated heparin was then injected (100 IU/kg) to obtain ACT>300. Complete IVC occlusion, 15 cm long, was confirmed upstream of the vena cava filter by angiography (Figure 2A). The occlusion was crossed by a 0.35 mm hydrophilic wire (Terumo, Japan) up to the right atrium (Figure 2B). Reopening was performed by repeated angioplasties with a 5.0x20mm noncompliant balloon at 8 atm (Mustang, Boston Scientific) involving all the occluded segment up to the vena cava filter (Figure 2C). Regarding the risk of thrombus embolization from thrombosed IVC during angioplasty, we performed continuous control of IVC hemodynamics and flow on TEE. The ASD was wired with hydrophilic wire and a 5‐French multipurpose launcher (Cordis) to position a nonhydrophilic 0.35 mm exchange wire (Medtronic) in the left superior pulmonary vein (Figure 2D). To limit crossing the vena cava filter, no ASD balloon calibration was performed. Prothesis sizing was based only on TEE, given the usual underestimation with ultrasound (on average, 15%‐20%). A 9‐French ASD sheath (Occlutech, Germany) was positioned in the left atrium. The Flex II 24 mm ASD Occluder (Occlutech, Germany) was deployed under TEE control (Figure 2E). Stability testing and edge trapping were perfect, allowing delivery. The final result was perfect, without residual shunt (Figures 1D and 2E). Final angiographic control confirmed IVC reopening without venous rupture (Figure 2F). Aspirin 250 mg was injected before removing the desilet. The complete procedure lasted 30 min. At 1 day, the ASD prosthesis was well positioned, without pericardial effusion on transthoracic ultrasound, and no IVC effusion or filter distortion on abdominal CT. The patient was discharged at 1 day. During clinical follow‐up at 3 months, no clinical events were observed. Considering the good tolerance, the absence identified of thrombophilic factors and the definitive contraindication against anticoagulation, after concertation, it was decided to leave the thrombosed IVC in situ.

## 3 DISCUSSION

This case shows, for the first time, the feasibility of using IVC access despite a thrombosed inferior vena cava filter for a cardiac procedure (here, ASD occlusion). The procedure, under local anesthesia, was quick and safe. The risks appeared to be very low: thrombus embolization could be feared during angioplasty, despite short anticoagulation by heparin, or else venous rupture, but neither occurred, probably thanks to the small diameter of the angioplasty balloon compared with the diameter of the vein.

Rates of vena cava filter implantation are decreasing, but specific indications remain, especially in stroke, and not only in case of suspected paradoxical embolism. In the literature, half of all patients admitted to hospital with acute stroke develop deep venous thrombosis and more than a quarter develop pulmonary embolism,^3^ usually without clinical symptoms. Given the risk of cerebral bleeding, an IVC filter is often the best prophylactic solution. As the IVC filter is frequently not removed, the risk of IVC thrombosis is increased and was 20% in one report.^4^ Many IVC reopening techniques were developed, mainly in case of clinical symptoms,^5^, ^6^ but most are complex, needing specific device implantation,^7^ with risk of complications such as venous rupture or IVC filter distortion, with loss of mechanical effect.^8^

In parallel, indications for percutaneous cardiac treatment after stroke have increased in recent years. New indications such as patent foramen ovale (PFO) occlusion ^9^ and new techniques such as left atrial appendage closure (LAAC) ^10^ have appeared. PFO occlusion and LAAC usually concern patients with history of stroke, in whom anticoagulation treatment is contraindicated in the acute phase, making IVC filter a useful option. PFO occlusion and LAAC systematically need femoral venous access. Thus, in the future, cases where a partially or totally thrombosed femoral vena cava filter needs to be crossed will probably become more frequent. The present case demonstrated the technical feasibility of easy and safe partial IVC reopening in the same step as the cardiac procedure.

## 4 CONCLUSION

Partially or completely thrombosed IVC filters should not be considered as contraindicating cardiac procedures with requiring femoral venous access. Simple IVC reopening by angioplasty can be performed in the same step as the cardiac procedure.

## CONFLICT OF INTEREST

None declared.

## AUTHOR CONTRIBUTIONS

We affirm that all individuals listed as authors agree that they have met the criteria for authorship and agree to the conclusions of the study and that no individual meeting the criteria for authorship has been omitted.

## ETHICAL APPROVAL

Informed consent was obtained from the patient regarding the report of her clinical scenario in an anonymous way.

## Data Availability

None declared.
